# The Integrin Activating Protein Kindlin-3 Is Cleaved in Human Platelets during ST-Elevation Myocardial Infarction

**DOI:** 10.3390/ijms20246154

**Published:** 2019-12-06

**Authors:** Bjoern F. Kraemer, Tobias Lamkemeyer, Mirita Franz-Wachtel, Stephan Lindemann

**Affiliations:** 1 Medizinische Klinik und Poliklinik I, Klinikum der Universität München, Marchioninistrasse 15, 81377 Munich, Germany; bjoern.kraemer@klinik-ebe.de; 2 Cluster of Excellence (CECAD) Cologne, Mass Spectrometry Facility at the Institute for Genetics, University of Köln, Josef-Stelzmann-Str. 26, 50931 Köln, Germany; 3 Proteasome Center Tuebingen, University of Tuebingen, Auf der Morgenstelle 15, 72076 Tübingen, Germany; 4 FB 20–Medizin, Philipps Universität Marburg, Baldingerstraße, 35032 Marburg, Germany; 5 Medizinische Klinik II, Klinikum Warburg, Hüffertstr. 50, 34414 Warburg, Germany; 6 Medizinische Klinik und Poliklinik III, Universitätsklinikum Tübingen, Otfried-Muller-Str. 10, 72076 Tübingen, Germany

**Keywords:** platelets, proteolysis, kindlin-3, myocardial infarction

## Abstract

Kindlins are important proteins for integrin signaling and regulation of the cytoskeleton, but we know little about their precise function and regulation in platelets during acute ischemic events. In this work, we investigated kindlin-3 protein levels in platelets isolated from patients with ST-elevation myocardial infarction (STEMI) compared to patients with non-ischemic chest pain. Platelets from twelve patients with STEMI and twelve patients with non-ischemic chest pain were isolated and analyzed for kindlin-3 protein levels and intracellular localization by immunoblotting and two-dimensional gel electrophoresis. Platelet proteome analysis by two-dimensional gel electrophoresis and protein sequencing identified kindlin-3 as a protein that is cleaved in platelets from patients with myocardial infarction. Kindlin-3 full-length protein was significantly decreased in patients with STEMI compared to patients with non-ischemic chest pain (1.0 ± 0.2 versus 0.28 ± 0.2, *p* < 0.05) by immunoblotting. Kindlin-3 showed a differential distribution and was primarily cleaved in the cytosolic and membrane compartment of platelets in myocardial infarction. Platelet activation with thrombin alone did not affect kindlin-3 protein levels. The present study demonstrates that kindlin-3 protein levels become significantly reduced in platelets of patients with myocardial infarction compared to controls. The results suggest that kindlin-3 cleavage in platelets is associated with the ischemic event of myocardial infarction.

## 1. Introduction

Kindlins are a group of intracellular proteins that have a central role in cellular adhesion and cell-matrix interactions. Three kindlin family members, kindlin-1, -2 and -3 have been identified which differ in tissue distribution [1]. Kindlins have emerged as elementary components for integrin signaling and activation and interact with cytoskeletal proteins like talin [2]. Kindlins also interact directly with β1, β2 and β3 integrins and contain FERM (Fermitin family) domains for interaction with extracellular matrix [3]. Especially kindlin-3 is expressed in hematopoietic cells, primarily platelets and megakaryocytes, and a lack of kindlin-3 expression results in compromised hemostasis and inflammation [4,5]. Kindlin-3 directly interacts with alpha(IIb)beta(3)-receptors during platelet aggregation [6], independently of talin [7]. Inherited kindlin-3 deficiency thus results in impaired platelet aggregation and bleeding, as well as osteopetrosis and leukocyte related immune deficiency due to impaired cell adhesion [4,7]. Uncontrolled bleeding in kindlin-3 deficient mice was due to impaired platelet integrin activation and led to early mortality within a few weeks [6,8]. Mutations or defects in interaction partners of kindlin induced a similar, yet less extreme disease phenotype as the kindlin defect. Glanzmann’s thrombasthenia, which is caused by direct mutations of alpha(IIb)beta(3)-integrins, is also characterized by prolonged bleeding. Likewise, mutations of kindlin-3 were identified as the cause of leukocyte adhesion defects [9]. During myocardial infarction platelet activation and aggregation are rapidly initiated and promote obstruction of diseased blood vessels [10,11]. Anti-aggregatory therapy that targets activation and integrin receptors of platelets has thus become a mainstay of therapy for myocardial infarction and has markedly improved patient survival [12,13]. Numerous markers of platelet activation have been studied in myocardial infarction, some of which correlate with disease severity [14,15,16,17]. Cleavage of kindlin-3 has been shown to be a mechanism to regulate cell shape changes in platelets [18]. We and others have previously demonstrated that cytoskeletal proteins such as talin-1 are regulated by proteolytic cleavage during platelet aggregation and shape change [19,20]. Although the central role of kindlins for integrin activation and platelet aggregate formation is well studied, kindlin protein levels in human platelets during myocardial infarction have not been investigated. Two-dimensional electrophoresis of platelet lysates from patients with myocardial infarction revealed increased kindlin-3 cleavage in patients with myocardial infarction. In contrast, kindlin-3 levels were unaffected in matched control platelets. Western blot analysis confirmed a significant decrease of full-length kindlin-3 protein and altered intracellular distribution of kindlin-3 in platelets of the myocardial infarction group. We provide the first evidence that kindlin-3 undergoes quantitative changes and processing, with distinct intracellular distribution in platelets during myocardial infarction, that could provide the basis for future investigations of this platelet phenotype in other ischemic conditions.

## 2. Results

### 2.1. The Proteome of Platelets from Patients with Myocardial Infarction Shows Distinct Changes

**Figure 1:**
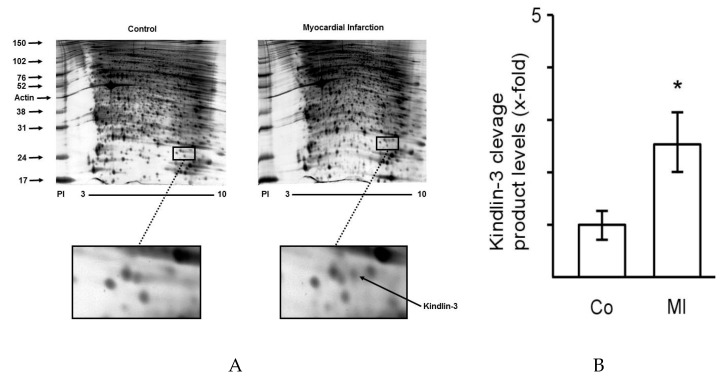
The platelet proteome displays distinct changes during myocardial infarction. (**A**) Two-dimensional gel electrophoresis of the platelet proteome demonstrates few distinct changes of protein expression during myocardial infarction (right) compared to platelets from patients with non-ischemic chest pain and without coronary artery disease (left). The area of differential protein regulation and identification of kindlin-3 is marked by boxes. The gel is representative of twelve independent patient pairs. The *Y*-axis indicates the protein size marker (kDa), the *x*-axis shows the pH range (PI). (**B**) Quantitative analysis of kindlin-3 cleavage by 2D-gel analysis in control patients (Co, *n* = 12) versus patients with myocardial infarction (MI, *n* = 12). Level of cleaved kindlin-3 in control patients was set as 1 and relative increase in protein cleavage is expressed as fold-increase versus control. * *p* < 0.05.

To identify protein targets that are differentially regulated in platelets during myocardial infarction, platelet lysates were analyzed by two-dimensional gel electrophoresis. Figure 1 shows platelet protein expression in platelets by 2D-gel electrophoresis from patients with myocardial infarction compared to matched controls with non-ischemic chest pain. Visibly few proteins are differentially regulated and show increases or decreases in protein quantity in myocardial infarction in comparison to patients with non-ischemic chest pain. Representative gels from a patient with ST-elevation myocardial infarction (right) and a control patient with non-ischemic chest pain (left) are shown. The region where differential protein regulation is visible and where kindlin-3 is located is marked by boxes. Clinical characteristics of control patients and patients with myocardial infarction are shown in Table 1.

### 2.2. Kindlin-3 Is Cleaved in Platelets during Myocardial Infarction

**Figure 2:**
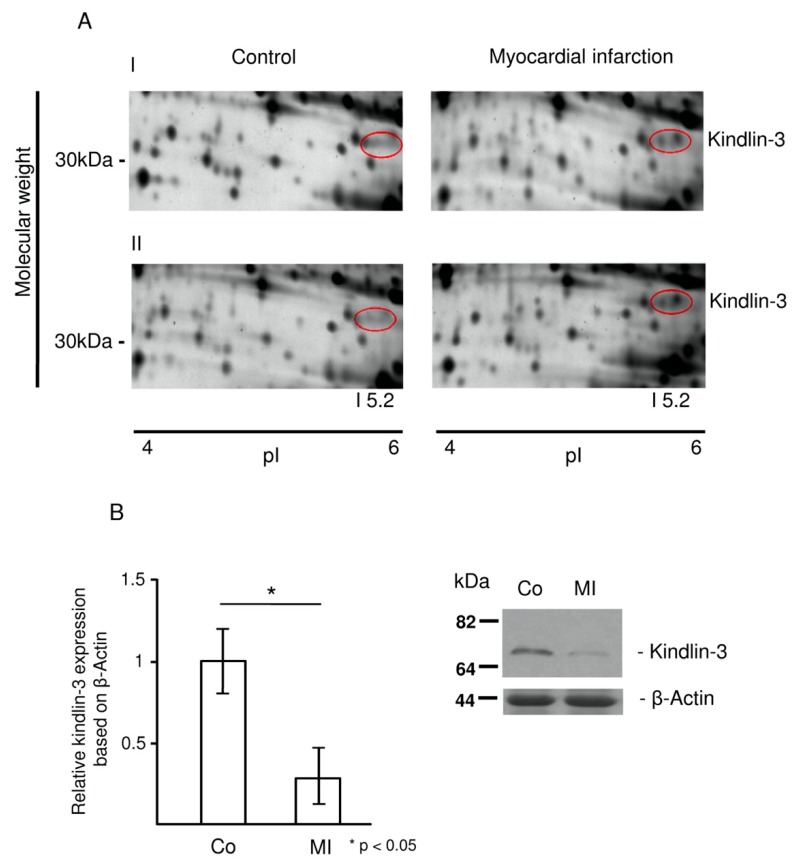
Kindlin-3 is cleaved in platelets during myocardial infarction. (**A**) Protein spots that represent fragments of kindlin-3 according to mass spectrometry appear in patients with ST-elevation myocardial infarction, whereas they are not detectable in patients with non-ischemic chest pain (red circles). Images show sample pairs of two patients with myocardial infarction and control (I and II) which are representative of twelve independent patient pairs. (**B**) Proteolytic processing of full-length kindlin-3 in platelets from patients with myocardial infarction (MI) was confirmed by Western blot. Quantitative analysis of kindlin-3 protein in the group of patients with myocardial infarction (*n* = 12) showed a significant decrease of full-length kindlin-3 protein levels compared to controls (*n* = 12); * *p* < 0.05. Mean kindlin-3 to actin ratio for the MI group was set as 1 and relative decrease in kindlin-3 protein in the control group was compared accordingly. A representative platelet sample pair from a patient with myocardial infarction (MI) illustrates the decreased full-length kindlin-3 protein (right lane) compared to a patient with non-ischemic chest pain (Co, left lane).

Protein spots of interest that were differentially regulated were sampled and analyzed by mass spectrometry. Figure 2A shows an example of new protein spots that appear in platelets from patients with myocardial infarction in two-dimensional gel electrophoresis. The double spot was identified as a cleavage product of kindlin-3 by mass spectrometry and was therefore found in the ~30 kDa region instead of the expected 75 kDa molecular weight region. Representative two-dimensional electrophoresis gels from two patients (I and II) with ST-elevation myocardial infarction and matched controls with non-ischemic chest pain are shown in Figure 2A. Western blot analysis using a specific anti-kindlin-3 antibody for full-length protein (75 kDa) showed a significant decrease of kindlin-3 protein in platelets from patients with myocardial infarction (1.0 ± 0.2 vs. 0.28 ± 0.2, * *p* < 0.05) (Figure 2B).

### 2.3. Kindlin-3 Cleavage Is not Induced by Thrombin Stimulation

**Figure 3:**
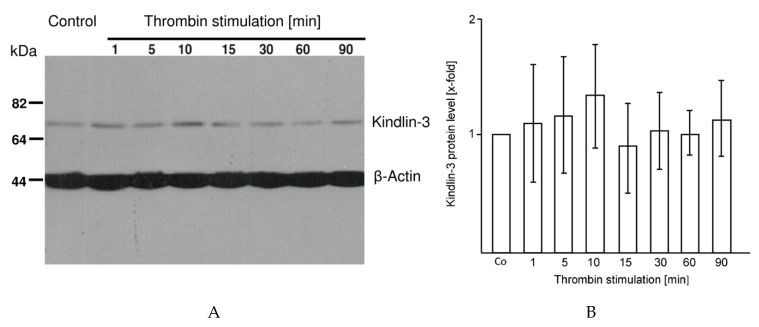
Cleavage of kindlin-3 is not a result of thrombin activation (**A**) Platelets (3 × 10^7^) were stimulated with thrombin (0.1 U/mL) for 1, 5, 10, 15, 30, 60 and 90 min and kindlin-3 levels were quantified by immunoblotting. Thrombin activation did not induce kindlin-3 degradation in platelets. β-actin served as loading control. The blot is representative of 4 independent experiments. (**B**) Quantitative analysis of kindlin-3 protein levels during thrombin stimulation. Mean kindlin-3 protein level of controls (Co) was set as 1 and protein levels after thrombin stimulation at time points 1, 5, 10, 15, 30, 60 and 90 min were compared (x-fold expression) and showed no differences.

In order to determine if cleavage of kindlin-3 in platelets during myocardial infarction was a result of thrombin activation, platelets were activated with thrombin (0.1 U/mL) for 1, 5, 10, 15, 30, 60 and 90 min. No quantitative differences in kindlin-3 protein were detectable with thrombin activation in repeated experiments (*n* = 4) (Figure 3A,B).

### 2.4. Kindlin-3 Is Located in the Cytoskeleton, the Plasma Membrane, and the Soluble Fraction of Human Platelets and Becomes Redistributed during Myocardial Infarction

**Figure 4:**
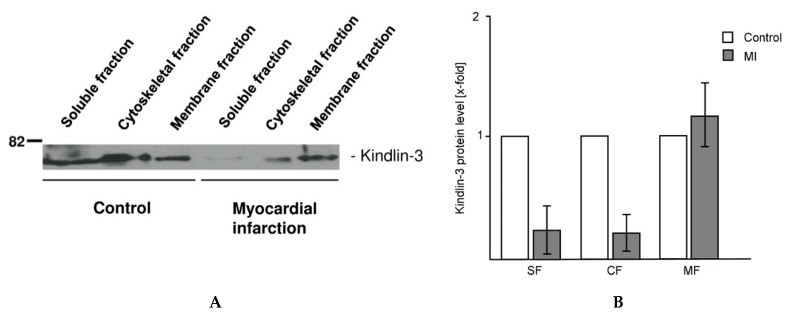
Intracellular distribution of kindlin-3 changes during myocardial infarction (**A**) Kindlin-3 protein levels in parts of the platelet that are associated with the soluble, cytoskeletal, and membrane fraction of platelets were analyzed by Western blot. The soluble protein fraction represents the cytoplasmic proteins. Lanes 1 to 3 represent cellular fractions of control platelets, lanes 4–6 fractions of platelets from myocardial infarction. Full-length kindlin-3 is reduced in the soluble and cytoskeletal fraction of platelets during myocardial infarction (lanes 4 and 5) whereas the membrane fraction is unaffected (lane 6). The blot is representative of twelve independent patient pairs. (**B**) Quantitative analysis of kindlin-3 protein levels in patients with myocardial infarction (MI) compared to control patients in the soluble fraction (SF), cytoskeletal fraction (CF), and membrane fraction (MF) of platelets. Mean kindlin-3 level of controls (Co) was set as 1 and quantitative changes are shown as x-fold expression versus control (x-fold).

To quantify intracellular kindlin-3 distribution, platelet lysates of patients with myocardial infarction and controls were separated into the soluble, cytoskeletal, and plasma membrane fraction by step-wise ultracentrifugation. Western blot analysis of the protein fractions revealed that proteolytic processing of kindlin-3 primarily occurred in those parts of the platelet that are associated with the soluble and cytoskeletal fractions isolated by ultracentrifugation, whereas the platelet structures that are associated with the membrane fraction showed no quantitative differences between the control and myocardial infarction groups (Figure 4A,B). Platelets from control patients showed a widely homogenous distribution of kindlin-3 in all cell fractions. Figure 4 shows a representative Western blot of twelve sample pairs.

### 2.5. Patient Characteristics

We have summarized clinical characteristics of control patients and patients with ST-elevation myocardial infarction in Table 1.

## 3. Discussion

In the present study, we analyzed protein levels of kindlin-3 in platelets from patients with acute myocardial infarction which we compared to matched control patients with non-ischemic chest pain. So far, levels of kindlin-3 in platelets have not been studied in acute myocardial infarction.

We demonstrated for the first time that protein levels of full-length kindlin-3 were significantly reduced in platelets of patients with acute myocardial infarction compared to patients with non-ischemic chest pain. We observed that kindlin-3 is cleaved to a 30 kDa fragment in platelets during myocardial infarction. There was no difference in antiplatelet therapy between groups by the time of blood sampling as control patients had all been treated with aspirin due to suspected coronary artery disease, and additional antiplatelet therapy in myocardial infarction patients was administered immediately after blood sampling to make sure that the observed effects could be attributed to the acute vascular event and not to drug-related effects.

Kindlin-3, which has become an interesting target to modulate integrin activation, could represent a so far unidentified platelet protein that is associated with acute vascular events. Interestingly, previous work found increased kindlin-3 expression in unstable plaque tissue prone to rupture, likely due to accumulation of platelets and monocytes [21]. Kindlins are a group of proteins that have been shown to be critically involved in platelet integrin activation, platelet aggregation, and interaction with extracellular matrix proteins. Therefore, defects in kindlin expression or function result in adhesion defects of tissue and blood cells. As a consequence, skin blistering, defective leukocyte adhesion, and impaired platelet aggregation are attributable to impaired kindlin function [4,22,23]. Especially in platelets, which perform elementary functions in hemostasis, defects in kindlin-3 and subsequently impaired integrin alpha(IIb)beta(3) activation have been observed [7]. Acute myocardial infarction is characterized by excessive platelet activation and aggregation which has become a central target for therapy. Several markers of platelet activation have been studied in platelets during acute myocardial infarction, including adhesion receptors such as GPVI [13] and pro-inflammatory mediators like SDF-1 [17]. In this context, it seems plausible that kindlin-3, which is directly connected to platelet integrin activity, shows measurable changes in acute myocardial infarction. Initially, we analyzed the platelet proteome during myocardial infarction and control platelets by two-dimensional gel electrophoresis, which led to the identification of new protein spots in the myocardial infarction group. Protein sequencing by mass spectrometry identified the new protein fragments as kindlin-3 cleavage products, which was initially a surprise as the proteins were found in the ~30 kDa region instead of the expected 75 kDa molecular weight area. Cleavage of other structural proteins such as filamin A and talin-1 has been shown to be a regulatory mechanism for platelet shape change during platelet activation [19,20]. A study by Zhao and colleagues reveals that cleavage of kindlin-3 by calpain controls the dynamics of integrin adhesion complexes [18]. Therefore, decreased full-length kindlin-3 levels and detection of kindlin-3 cleavage products in platelets in the process of myocardial infarction makes perfect sense. Cleavage products were not detected by immunoblotting because the target epitope that is recognized by the detection antibody was no longer intact, but cleavage products of kindlin-3 were consistently detected on 2D gels. Microparticle release is also considered to be a result of degradation or cleavage of structural proteins (e.g., talin) by intracellular proteases like calpain, as shown in hepatocytes [24]. Platelet microparticle release is also mediated through calpain activation, although the precise mechanisms are not fully understood [25]. In previous work, we were able to identify protein degradation of the antiapoptotic protein Bcl-xL by calpain as a mechanism for programmed cell death [26]. It appears likely that more platelet functions and processes are regulated through selective proteolysis of structural or regulatory proteins. Interestingly, our data show that cleavage of kindlin-3 occurs in selected protein fractions, which likely depends on the intracellular distribution of proteases that cleave kindlin-3 protein. We observed proteolytic processing of kindlin-3 preferentially in the soluble and cytoskeletal fraction of platelets from patients with myocardial infarction whereas the membrane fraction was unaffected. Kindlin-3 protein levels were further unaffected by thrombin stimulation which underscores that the underlying mechanism is not purely activation-dependent. It thus seems likely that the intracellular localization of kindlins may further change based on cellular function and receptor activation. Previous work has also speculated that kindlins enable cells to rearrange their cytoskeleton and to enable migration [27,28]. Especially kindlin-3 is present in podosomes and integrin adhesion sites in migrating cells [1,29]. Interestingly, proteolytic cleavage of kindlin-3 by calpain was also identified as one of the processes that control the adhesion dynamics that enable leukocytes to migrate [18]. We have previously been able to demonstrate the capacity of platelets to migrate along an SDF-1 gradient and to transmigrate into inflamed vessel walls [30,31]. This involves activation of cytoskeletal regulators and rearrangement of the cytoskeleton for focal adhesion formation [32]. It thus seems possible that kindlins may play a role in both platelet adhesion and platelet migration. Overall, it remains to be determined if cleavage of kindlin-3 in platelets during acute myocardial infarction is a result of the disease process itself or if its differential regulation during platelet aggregation affects acute vascular events. Mutations of kindlin-3 can result in antithrombotic effects as previously described [7], which may also occur with differential proteolytic cleavage of kindlin-3. In this work, we demonstrate for the first time that kindlin-3, which is paramount for integrin activation, is cleaved in platelets during myocardial infarction following a reproducible intracellular distribution. Due to its physiological role in cytoskeletal regulation and its distinct protein phenotype during myocardial infarction in platelets, kindlin-3 could be an interesting platelet marker in vascular ischemia. Whether this phenotype is prothrombotic or whether it is a stress response to the ischemic environment remains to be determined. Further research will be necessary to elucidate the complex mechanisms of kindlin functions in platelets in vitro and under acute ischemic conditions. The observed platelet phenotype described in this study lays the groundwork for future investigations and it will be interesting to also extend the observed association of kindlin-3 cleavage in myocardial infarction to other acute ischemic conditions such as stroke or peripheral artery disease.

## 4. Material and Methods

### 4.1. Study Design

Twelve patients with acute ST-elevation myocardial infarction (STEMI) and obstruction of a proximal left dominant or right coronary artery as well as twelve age-matched control patients which presented with typical chest pain but showed no coronary artery disease in coronary angiography were enrolled in the study. Myocardial infarction was eventually ruled out by electrocardiogram and serially negative cardiac enzymes in control patients. Patients with ST-elevation myocardial infarction were treated with aspirin immediately after diagnosis. Blood for this study was drawn in the cath lab before other antiplatelet agents such as clopidogrel or tirofiban were administered for acute myocardial infarction. All control patients were also on aspirin treatment by the time of blood sampling due to acute onset chest pain and suspected coronary artery disease. Clinical characteristics of patients are shown in Table 1. Written informed consent was obtained from all patients. The study was approved by the local ethics committee of the University of Tübingen (No. 264/2007BO2, approved 25 October 2007) in accordance with the Declaration of Helsinki. Blood was drawn on admission after indication for coronary angiography had been established and blood samples were processed immediately.

### 4.2. Platelet Isolation

Platelets were isolated according to standard protocols in our lab as previously described [20,30,33]. In brief, whole blood was drawn directly into plastic tubes containing sodium citrate (1:10). After centrifugation of the whole blood without brake at 340× *g* for 15 min at room temperature (RT), the platelet-rich plasma (PRP) was carefully removed. Platelets in PRP were pelleted at 600× *g* for 10 min at RT and carefully resuspended in warm M199 culture media. For some experiments, platelets (1 × 10^9^) were stimulated with thrombin (Sigma-Aldrich, Taufkirchen, Germany) 0.1 U/mL for 1, 5, 10, 15, 30, 60 and 90 min prior to lysis and at −20 °C.

### 4.3. Fractionation of Soluble, Cytoskeleletal and Platelet Membrane Proteins

Freshly isolated platelets (1 × 10^9^) were lysed in 1 mL Triton X-lysis buffer (containing Complete protease inhibitor, Roche, Penzberg, Germany) for 10 min on ice. The pellets containing the cytoskeletal cell fraction were collected after centrifugation at 15,600× *g* for 4 min, after the supernatants had been carefully removed. Supernatants were centrifuged again at 100,000× *g* and pellets containing the membrane fraction were collected. Soluble proteins were concentrated from the supernatant by acetone precipitation. All fractions were resuspended in SDS loading buffer before electrophoresis and immunoblotting was performed as described below.

### 4.4. Two-Dimensional Gel Electrophoresis

Platelets from patients with myocardial infarction and non-ischemic chest pain were lysed in 100 µL of CHAPS lysis buffer (8M Urea, 4% CHAPS, 2% DTT) and purified using a 2D Clean-up kit (GE Healthcare, Freiburg, Germany) according to the manufacturer’s instructions. Comparative 2D gel analysis of the proteomes was performed as described previously with slight modifications [34]. First dimension isoelectric focusing was performed using a Protean IEF Cell focusing unit (BioRad, Hercules, CA, USA) with pH 3–10NL gel strips (11 cm, GE Healthcare, for some subsequent gels pH 4–7). For the second dimension, equilibrated (5.7M Dithiothreitol and 1.5M Iodoacetamide) gel strips were applied to 12% polyacrylamide gels. Proteins were stained with Lava purple fluorescent staining (Fluorotechnics, Sydney, Australia). Protein spots of interest were excised manually from the gels, digested with trypsin and analyzed by LC ESI-MS/MS as described previously [35]. Peptide sequence analysis by mass spectrometry of the isolated protein spots resulted in the identification of the differential cleavage of kindlin-3 in the myocardial infarction group.

### 4.5. Immunoblotting

Platelets (1 × 10^9^) lysed in Laemmli buffer were separated by SDS PAGE and stained for kindlin-3. After protein transfer to a nitrocellulose membrane, membranes were blocked in 5% nonfat milk in TBS-T for 1 h. Afterward, primary antibody to full-length kindlin-3 (1:100 in 3% nonfat milk/TBS-T, Abcam, Cambridge, UK) was added and incubated overnight at 4 °C with constant agitation. Membranes were washed in TBS-T repeatedly and HRP-conjugated secondary antibody (1:10,000, GE Healthcare, Freiburg, Germany) was added for 1 h at room temperature. After washing, chemiluminescent substrate (ECL reagent, Amersham Biosciences, Munich, Germany) was added for 1–5 min and bands were visualized on plain film. β-Actin served as loading control (rabbit anti-human β-Actin, Cell Signaling Technology, Dallas, TX, USA). Kindlin-3 protein levels were quantified by band densitometry analysis with the help of a kindlin-3 to actin ratio. 

### 4.6. Statistical Analysis

All data are presented as means ± SEM. Statistical analyses were performed with Sigma Plot 10.0. For comparisons between two groups of normally distributed data, the student’s t-test was used. For the comparison of two groups without normally distributed data, a rank-sum test was performed. For multiple comparisons between groups of normally distributed data, the one-way analysis of variance (one-way ANOVA) was used. Differences were considered significant at an error probability level of *p* < 0.05.

## Figures and Tables

**Table 1:** Clinical characteristics of 12 patients with ST-elevation myocardial infarction (STEMI) and 12 age matched control patients are shown. Cardiovascular risk factors such as arterial hypertension, diabetes mellitus, and smoking, as well as renal function are listed. Numbers indicate *n* of 12 with positive baseline characteristics in each group. Impaired renal function was defined as an elevated serum creatine level above 1.2 mg/dL.

	Control Group (*n* = 12)	Myocardial Infarction (STEMI) (*n* = 12)
Age (years, mean ± SEM)	65 ± 3	67 ± 4
Arterial Hypertension; *n*=	6	7
Diabetes mellitus; *n*=	3	2
Smoking; *n*=	6	7
Impaired renal function; *n*=	2	2
